# Carvacrol Reduces Virulence Traits in *Meyerozyma guilliermondii* and *Candida dubliniensis* and Enhances *Galleria mellonella* Survival During *Candidozyma auris* Infection

**DOI:** 10.3390/microorganisms14010188

**Published:** 2026-01-14

**Authors:** Andrea Giammarino, Laura Verdolini, Javier Mussin, Giulia Radocchia, Florencia Rojas, Gustavo Giusiano, Letizia Angiolella

**Affiliations:** 1 Department of Public Health and Infectious Diseases, Sapienza University of Rome, Piazzale Aldo Moro 5, 00185 Rome, Italy; andrea.giammarino@uniroma1.it (A.G.); laura.verdolini@uniroma1.it (L.V.); giulia.radocchia@uniroma1.it (G.R.); 2 Departamento Micología, Instituto de Medicina Regional, Universidad Nacional del Nordeste, CONICET, Av. Las Heras 727, Resistencia H3500, Chaco, Argentina; javiermussin@hotmail.com (J.M.); florenciarojas@hotmail.com (F.R.); gustavogiusiano@yahoo.com.ar (G.G.)

**Keywords:** *Candidozyma auris*, *Meyerozyma guilliermondii*, *Candida dubliniensis*, biofilm, adherence, hydrophobicity, osmotic stress, carvacrol, *Galleria mellonella*

## Abstract

Background: Antifungal resistance among *Candida* species and related genera, coupled with the lack of new drugs, poses a significant threat to public health. Several studies have demonstrated a relationship between virulence factors and resistance. Current objectives include identifying new targets and searching for new natural molecules. Carvacrol, a natural phenolic compound, has been shown to have antimicrobial properties; however, its impact on the virulence of species other than *Candida albicans* and related yeast genera remains underexplored. Methods: The antifungal activity of carvacrol was evaluated against clinical isolates of *Candidozyma auris*, *Meyerozyma guilliermondii*, and *Candida dubliniensis*, as well as its effect on adhesion, hydrophobicity, biofilm formation and osmotic stress tolerance. In vivo activity was assessed using the *Galleria mellonella* infection model at MIC concentrations. Results: Carvacrol inhibited adherence and significantly reduced both early and preformed biofilms in *M. guilliermondii* and *C. dubliniensis*. In *C. auris*, the compound produced a modest reduction in biofilm activity but significantly enhanced larval survival in the in vivo model (~20%, *p* < 0.01). Carvacrol also induced increased tolerance of *C. auris* to osmotic stress, suggesting activation of adaptive pathways. Conclusions: Carvacrol exhibits species-specific effects, acting as an antivirulence modulator in *M. guilliermondii* and *C. dubliniensis* and attenuating virulence in vivo in *C. auris*. These findings support the potential of carvacrol as an adjuvant antifungal strategy, particularly against *C. auris*, and highlight the relevance of targeting virulence traits to reduce selective pressure and limit antifungal resistance.

## 1. Introduction

*Candida* and other recently reclassified related genera have shown an increase in the number of cases causing fungal infections. The genus *Candida* includes more than one hundred different species, of which only a minority are involved in human infections. In fact, about 65% of *Candida* species do not grow at 37 °C, which prevents them from acting as pathogens or being identified as common components of the human microbiota [1]. Approximately 30–35% of candidiasis cases are currently caused by non-*albicans Candida* (NAC) species [2]. Some studies in an animal model show that NAC species are less virulent than *C. albicans*, while in humans their infectivity is equal, if not in some cases higher [3].

*Candidozyma auris* is known for its ability to cause invasive infections, particularly in people with compromised immune systems, such as hospitalized patients or those with chronic diseases [4]. Since its first identification, there has been an exponential increase in reports of infections and outbreaks in healthcare settings around the world. A concerning feature of *C. auris* is its resistance to several classes of antifungal drugs. In addition, it exhibits several worrying characteristics compared to other *Candida* species: persistent colonization of skin and hospital surfaces, ability to resist common disinfectants, and rapid spread among patients [5]. Some reports describe the persistence of *C. auris* on the skin despite daily cleansing with chlorhexidine [6,7]. The strong persistence of *C. auris* on abiotic hospital instrument surfaces has been correlated with the occurrence of Intensive Care Unit (ICU) outbreaks. The biofilm produced by *C. auris* is able to resist commonly used disinfectants, including quaternary ammonium compounds [8]. The origin of *C. auris* is unclear, and it is impossible to determine whether environmental and climate changes have contributed to its recent emergence as a pathogen [9].

*Candida dubliniensis* is an opportunistic pathogen causing infections in immunocompromised patients with the ability to rapidly develop resistance to fluconazole in vitro. This species is phenotypically and genetically closely related to *C. albicans* [10,11]. Although *C. albicans* and *C. dubliniensis* share similar phenotypic characteristics, several studies have suggested that *C. dubliniensis* is less virulent than *C. albicans*, with the best-described virulence factors being adhesins, genes involved in biofilm formation, immune evasion, thermotolerance, and phospholipases and proteinases [12].

*Meyerozyma guilliermondii* is a complex of species, saprophytes on human mucosa and skin, including *M. guilliermondii sensu stricto*, *M. fermentati* and *M. carpophila*. *M. guilliermondii* species complex has been increasingly reported to cause serious infections in individuals with risk factors and is characterized by reduced susceptibility to azoles and echinocandins [13]. *M. guilliermondii* produces biofilms with low metabolic activity and moderate biomass [13], as well as phospholipase C and D and aspartic protease [14].

Antifungal resistance due to the increasing number of multidrug-resistant microorganisms and the unavailability of new antifungal agents is recognized as one of the most important public health threats of the 21st century [15,16]. Acquired antifungal resistance is associated with activating the stress response as an adaptive mechanism, altering drug targets, or overexpressing and increasing multidrug transporters [17]. This emergence has urged researchers to explore therapeutic alternatives, one of which includes the use of natural products such as essential oils (EO) [18] or their components with antifungal activity.

Carvacrol is a monoterpene phenol and a major component of EO extracts from oregano and other *Labiatae* plants. It is nontoxic for humans and has pharmacological properties, including anti-inflammatory, anticancer and antidepressant (IC_50_ of 105 μg/mL) [19]. In a previous study, we demonstrated that carvacrol was able to inhibit the virulence factors, such as hydrophobicity, adherence and biofilm formation of *M. sympodialis* and *M. furfur*, two important species involved in cutaneous infections [20]. We also proved a close correlation between the structural architecture of cell walls and the organization of biomembranes with the fungal cell response to carvacrol treatment in *Candida* spp. [21]. Many studies have reported that carvacrol is capable of reducing bacterial growth and biofilm formation [22,23,24,25]. The antifungal and antibiofilm activity of carvacrol has also been evaluated against certain *Candida* species [26].

Drug resistance in *Candida albicans* can be linked to several virulence factors. Indeed, the acquisition of resistance to echinocandins or fluconazole was associated with a selective increase in experimental pathogenicity for systemic infection of an originally poorly virulent *C. albicans* strain, which exhibits massive hyphal formation in the organs [27]. The most important virulence factors in *C. albicans* are adherence, dimorphism, biofilm formation, and the production of hydrolytic enzymes (such as proteases, lipases and haemolysins) as well as toxins [28,29]. Adherence is essential for fungal colonization and spread, as it contributes to the persistence of the organism in the host [30]. Regardless of the species, microbial adherence through hydrophobic interactions depends on the hydrophobicity of the microbial surface [31]. Similarly, biofilm formation is an important virulence trait that supports fungal pathogenesis. Multicellular biofilms allow yeast cells to anchor to host tissues, catheters, implants, and other devices [32].

Using naturally occurring substances could be a significant step forward in finding new ways to overcome drug resistance. This study aims to evaluate the antifungal activity of the monoterpene carvacrol, and its effects on adhesion, biofilm formation and osmotic stress resistance, against *C. auris*, *C. dubliniensis* and *M. guilliermondii*, using both in vitro assays and the *Galleria mellonella* in vivo infection model.

This is the first study to examine the impact of carvacrol on *C. auris*. Data on *M. guilliermondii* and *C. dubliniensis* are scarce, and no previous study has evaluated the modulation of their virulence in the presence of carvacrol. This study is the first to demonstrate that carvacrol enhances larval survival in a *G. mellonella* infection model with *C. auris*. Additionally, we examined the effect of carvacrol on fungal adaptation to osmotic stress, an important yet understudied virulence factor.

## 2. Materials and Methods

### 2.1. Microorganisms and Growth Conditions

*Candidozyma auris* (IMR-M-L 1304) and *Meyerozyma guilliermondii* (IMR-M-L 1511) isolates from blood, and *Candida dubliniensis* (IMR-M-L 1510) standard strain were studied. The isolates were identified using the MALDI-TOF MS (matrix-assisted laser desorption ionisation–time of flight mass spectrometry) system (Bruker Daltonics, Bremen, Germany) and deposited in the culture collection (IMR-M-L) of the Mycology Department at the Instituto de Medicina Regional, Universidad Nacional del Nordeste, Argentina. This study was approved by the Ethics and Research Committee of the Instituto de Medicina Regional, Universidad Nacional del Nordeste, Argentina (Renis CE000326). All yeast isolates were grown on Sabouraud Dextrose Agar (SDA) (Biolife Italiana Srl, Monza, Italy) at 35 °C prior to the study of the virulence factors.

### 2.2. Materials

Polystyrene plates with 6, 24 and 96-wells (Corning Incorporated, Corning, NY, USA). Hamilton syringe 701N (volume 10 μL), needle size 26 s, cone tip, Petri dishes (100 mm diameter) from Sigma-Aldrich, Milan, Italy.

SDA culture medium was purchased from Biolife (Biolife Italiana Srl, Monza, Italy). Roswell Park Memorial Institute (RPMI 1640) medium, 3-(N-morpholino)-propane sulfonic acid (MOPS) buffer, Tween 20, dimethyl sulfoxide (DMSO), fetal bovine serum (FBS), carvacrol, fluconazole, ethanol, acetone, Dulbecco’s Phosphate Buffered Saline (PBS), 2-Methyl-1,4-naphthoquinone (menadione), 2,3-Bis(2-methoxy-4-nitro-5-sulfophenyl)-2H-tetrazolium-5-carboxanilide inner salt (XTT) were obtained from Merck KGgaA (Darmstadt, Germany).

### 2.3. Minimum Inhibitory Concentration (MIC)

The antifungal activity of carvacrol and fluconazole (used as the control) against all isolates was evaluated using the broth microdilution method proposed by the Clinical and Laboratory Standards Institute (CLSI) in reference CLSI M27-A4 [33]. The MIC was established by performing serial fold dilutions of carvacrol and fluconazole in RPMI 1640, supplemented with 3.4% MOPS and Tween 20 (final concentration: 0.01% *v*/*v*). Fluconazole dilutions ranged from 0.062 to 64 µg/mL while carvacrol dilutions ranged from 6 to 1560 µg/mL. The inoculum size was approximately 2.5 × 10^3^ cells/mL. The 96 well plates containing 200 μL per well were incubated at 35 °C for 24 h. The MIC was determined as the lowest concentration where no visible fungal growth was observed.

### 2.4. Cellular Surface Hydrophobicity (CSH) in Presence of Carvacrol

CSH was measured using a two-phase system as reported [34]. Yeast was cultivated in SD broth at 35 °C for 24 h, washed with sterile Phosphate Buffered Saline (PBS) containing 0.5% Tween 20, and resuspended in 0.05 M PBS (pH 7.2) at a concentration of 2 × 10^6^ cells/mL. The suspension was transferred to a glass tube with or without a MIC concentration of carvacrol and incubated for 1 h at room temperature. The cells were washed and resuspended in 5 mL PBS, then 500 µL octane was added. The mixture was vortexed for 1 min and left at room temperature to allow phase separation. The turbidity of the aqueous phase was measured at an optical density (OD) of 600 nm. The group without the octane overlay was used as the control. Relative CSH was calculated as follows: [(OD600 control − OD600 after octane)/OD600 control] × 100. Each value represents the mean of three independent biological replicates.

### 2.5. Adherence on Plastic Surface by Crystal Violet Assay in Presence of Carvacrol

The ability of yeast cells to adhere to a polystyrene surface was measured as described previously [35]. Yeasts were cultivated for 24 h at 35 °C, washed twice with sterile 0.5 M PBS pH 7.2, and resuspended at 37 °C in RPMI 1640 containing 10% FBS with or without a MIC concentration of carvacrol. The cell concentration was 2.5 × 10^7^ cells/mL. After 3 h of incubation at 37 °C in six-well plates, the medium was aspirated; and non-adherent cells removed by washing with PBS. Adherent cells were fixed with 99% methanol for 15 min and stained with 0.02% (*v*/*v*) crystal violet for 20 min. The cells were then washed, then 1 mL of 33% (*v*/*v*) acetic acid was added for 30 min. The dissolved crystal violet was measured at 590 nm. Adherence (%) was calculated as: [OD590 sample/OD590 control].

### 2.6. Early and Preformed Biofilm in Presence of Carvacrol

Biofilm formation was assessed as reported previously [36]. A yeast suspension (1.0 × 10^7^ cells/mL) was incubated for 3 h (early biofilm) or 24 h (preformed biofilm) at 37 °C in 24-well plates. Cells were then incubated with or without MIC concentration of carvacrol, and re-incubated for 24 and 48 h at 37 °C. The medium was aspirated and non-adherent cells removed by washing with sterile PBS. Biofilm development was measured using a 2,3-bis-(2-methoxy-4-nitro-5-sulfo-phenyl)-2H-tetrazolium-5-carboxanilide (XTT) reduction assay, which is a reaction catalyzed by mitochondrial dehydrogenases. Cells were washed with PBS, incubated with 0.5 mg/mL XTT and 1 µM menadione in PBS at 37 °C for 2 h, and 500 µL of the reaction solution was transferred to a fresh plate. XTT reduction was measured at OD 490 nm.

### 2.7. Infection Model in Galleria mellonella

*Galleria mellonella* larvae were used to evaluate microbial virulence and the efficacy/safety of antimicrobial agents [37,38]. Ethical approval was not required.

For survival analysis, 10 larvae were used for each yeast isolate and for three control groups (untreated larvae, larvae treated with physiological saline and larvae treated with 0.01% Tween 20 in physiological saline). All larvae had an average weight ± SD of 283.6 ± 2.7 mg/each. Figure S1 shows weight distribution histograms for each group of larvae, which range from 260 mg to 320 mg. Larvae previously incubated at 37 °C in aerobic conditions, were randomly selected and injected with 10 μL of yeast suspension (about 5 × 10^7^ cells CFU/mL) into the hemocoel through the last left proleg [39]. After inoculation with each species into the hemocell through the penultimate left leg, a MIC concentration of carvacrol in physiological saline with 0.01% Tween 20 was administered. Larvae were then placed in Petri dishes at 37 °C and monitored every 24 h for 7 days. Larvae were considered dead when they showed no movement in response to gentle prodding. One control group was not injected; another was injected with physiological saline solution. Each experiment was repeated three times.

### 2.8. Sensitivity to Osmotic Stress in Sodium Chloride in Presence of Carvacrol

Sensitivity to NaCl was determined using the method reported by Chaves and da Silva [40], with modifications. Ten microlitres of yeast cells 1 × 10^4^ grown in SD medium at 35 °C for 24 h were transferred to a 96-well plate containing 100 µL SD broth, with or without an MIC concentration of carvacrol, and increasing NaCl concentrations ranging from 0.03% to 30%. Plates were incubated at 35 °C for 48 h, and growth in the presence of NaCl was assessed.

### 2.9. Cell Line Cultivation and Sensitivity to Carvacrol

Carvacrol toxicity was evaluated using the T24, human urinary bladder carcinoma purchased from the American Type Culture Collection cell line (ATCC^®^ HTB-37™, Rockville, MD, USA) stored in liquid nitrogen. T24 cells were cultured in RPMI, with 10% FBS, 2 mM glutamine, 200 U/mL penicillin, and 200 µg/mL streptomycin at 37 °C in 5% CO_2_. To assess toxicity, the MTT (3-(4,5-dimethylthiazol-2-yl)-2,5-diphenyltetrazolium bromide) assay was performed. In a 96-well plate, 200 μL of cell suspension 2 × 10^5^ cell/mL were added well incubated at 37 °C, 5% CO_2_. After a 3 h or 24 h, the medium was replaced with RPMI and FBS containing MIC concentrations of carvacrol (48 and 390 μg/mL). Controls included untreated cells and cells treated with Tween 20, at the same concentration as in the carvacrol solution. After treatment, the medium was replaced with 100 μL of RPMI without FBS containing MTT 0.5 mg/mL final concentration. Plates were incubated 4 h at 37 °C, then the solution was removed and 100 μL DMSO were added to dissolve formazan crystals. Absorbance was read at 570 nm, reference 620 nm, using Tecan Sunrise microplate reader, (Tecan Group Ltd., Männedorf, Switzerland) [41].

### 2.10. Statistical Analysis

Statistical differences between groups were analyzed using Student’s *t*-test to account for variability in small samples. For survival rates, the log-rank (Mantel-Cox) test was used [42]. A *p* value ≤ 0.05 was considered significant. Analyses were performed using Graphpad Prism Software version 8.0.1.

## 3. Results

### 3.1. Antifungal Activity of Fluconazole and Carvacrol

Table 1 shows the antifungal activity of carvacrol against *C. auris*, *M. guilliermondii* and *C. dubliniensis*. Fluconazole was used as a control. Although the CLSI has established MIC breakpoints for several common *Candida* species, breakpoints for *C. auris*, *M. guilliermondii* and *C. dubliniensis* have not been defined yet [33]. *C. dubliniensis* was the most susceptible to fluconzole with a MIC value of 0.5 µg/mL., while less susceptibility was observed for *C. auris* and *M. guilliermondii*, MIC values being 4 and 32 µg/mL, respectively. The MIC values of carvacrol ranged from 48 to 390 μg/mL in the three tested species.

### 3.2. Adherence and Hydrophobicity in Presence of Carvacrol

**Figure 1:**
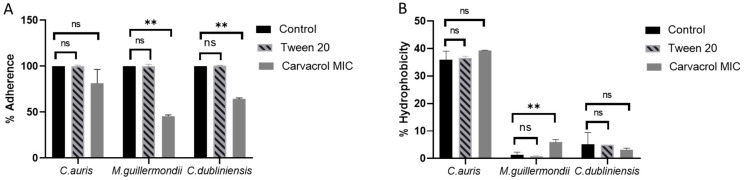
Histograms of % adherence and % hydrophobicity with and without MIC concentrations of carvacrol in *Candidozyma auris*, *Meyerozyma guilliermondii* and *Candida dubliniensis*. Control: cells not treated; Tween 20: cells treated with 0.01% of Tween 20; Carvacrol MIC: cells treated with MIC concentration of carvacrol: *C. auris* 48 μg/mL, *M. guilliermondii* 390 μg/mL and *C. dubliniensis* 390 μg/mL. (**A**) Percentage of adherence after 3 h of adhesion on plastic surface, evaluated by CV assay at 590 nm and calculated as follows: OD590 of the sample/OD590 of the control. (**B**) Percentage of hydrophobicity evaluated after 1 h of incubation by two-phase system at 600 nm. Relative CSH was calculated as follows: [(OD600 of the control − OD600 after octane overlay)/OD600 of the control] × 100. The results represent means ± standard deviation of three independent experiments. *p*-values were obtained using a Student’s *t*-test (** *p* < 0.01), ns = not significant *p* > 0.05.

Figure 1 shows the adherence (panel A) and hydrophobicity (panel B) histograms of *C. auris*, *M. guilliermondii* and *C. dubliniensis* in the presence of the carvacrol MIC concentration.

All clinical isolates showed strong adherence to abiotic surfaces, and no significant differences were observed in the controls. However, differences in inhibition were observed in the presence of carvacrol. In *C. auris*, the percentage of adherence obtained from different samples decreased from 100 ± 2.0% to 81.2 ± 15%, with a slight inhibition of 18.8 ± 5.6% compared to the control. Carvacrol significantly reduced adherence in *M. guilliermondii* and *C. dubliniensis*. In *M. guilliermondii*, the percentage of adherence in the control group was 100 ± 1.1%, whereas in the presence of carvacrol it was 45.5 ± 1.4%, representing an inhibition of 54.6 ± 1.7% (* *p* < 0.001). In *C. dubliniensis*, the percentage of adherence was 100 ± 0.1%, decreasing to 64,4 ± 1.0% in the presence of carvacrol, representing an inhibition of approximately 35.6 ± 2.6% (* *p* < 0.001) (Figure 1A).

The evaluation of hydrophobicity is presented in Figure 1B. *C. auris* showed about 39.25 ± 0.12% hydrophobicity in the control group, whereas the other species exhibited low levels of hydrophobicity 1.52 ± 1.0% and 5.17 ± 2.2% for *M. guilliermondii* and *C. dubliniensis* respectively. When carvacrol was added to *C. auris* and *C. dubliniensis*, no significant (*p* > 0.05) difference was observed. However, an increase in hydrophobicity was observed in *M. guilliermondii* 5.92 ± 0.92 (** *p* < 0.001).

### 3.3. Early Biofilm and Preformed Biofilm in Presence of Carvacrol

**Figure 2:**
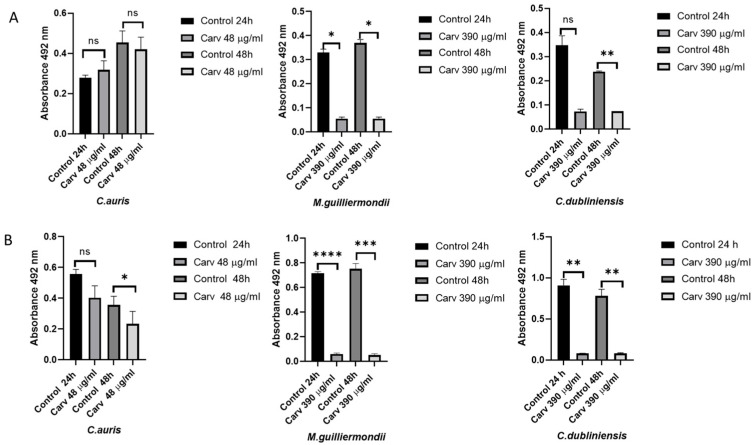
Early biofilm, after 3 h (**A**) and preformed biofilm after 24 h (**B**) in presence of carvacrol at MIC concentration after 24 h and 48 h of incubation at 37 °C in *Candidozyma auris*, *Meyerozyma guilliermondii* and *Candida dubliniensis*. Control: cells untreated; Carv: MIC concentration of carvacrol used to treat biofilm: *C. auris* 48 μg/mL, *M. guilliermondii* 390 μg/mL and *C. dubliniensis* 390 μg/mL. The results represent means ± standard deviation of three independent experiments. *p*-values were obtained using a Student’s *t*-test (* *p* < 0.05, ** *p* < 0.01, *** *p* < 0.001, **** *p* < 0.0001, ns = not significant *p* > 0.05).

Figure 2 shows the average readings obtained after 24 and 48 h for the early (Figure 2A) and preformed (Figure 2B) biofilms developed by the clinical isolates of *C. auris*, *M. guilliermondii* and *C. dubliniensis*, in both the presence and absence of carvacrol MIC concentrations. All clinical isolates produced biofilm with no significant differences at 24 and 48 h in the early biofilm stage. In the presence of carvacrol, a significant reduction of about 80 ± 2.4% was evident within the first 24 h, particularly among the *M. guilliermondii* and *C. dubliniensis* isolates (* *p* < 0.05) (Figure 2A), with OD492 values as low as 0.055 ± 0.007 and 0.075 ± 0.001, respectively. No changes were observed in *C. auris*, where the values remained similar to the control group (0.31 ± 0.04).

After 48 h, the inhibition was similar. *C. auris* biofilms were not significantly inhibited, while both *C. guilliermondii* (* *p* < 0.05) and *C. dubliniensis* (** *p* < 0.01) were inhibited by approximately 85 ± 1.3% and 70 ± 0.35% respectively (Figure 2A).

In all clinical isolates an increase in preformed biofilms was observed after 24 h and 48 h, but *M. guilliermondii* and *C. dubliniensis* showed higher values of preformed biofilms than *C. auris*. When carvacrol was added to the preformed biofilms, it reduced the *C. auris* biofilm by approximately 28 ± 10.0% within the first 24 h, although this difference was not significant (Figure 2B), with values ranging from 0.35 to 0.05. Interestingly, inhibition was approximately 91 ± 1.8% in both species *M. guilliermondii* (**** *p* < 0.0001) and *C. dubliniensis* (** *p* < 0.01).

After 48 h, a significant inhibition of around 33% ± 2.5 (* *p* < 0.05) with OD 492 values from 0.356 ± 0.06 to 0.233 ± 0.081 was observed in *C. auris*. The levels of inhibition remained at 91 ± 1.6%for *M. guilliermondii* (*** *p* < 0.001) and *C. dubliniensis* (** *p* < 0.01) (Figure 2B).

### 3.4. Survival of Galleria mellonella Larvae After Infection

**Figure 3:**
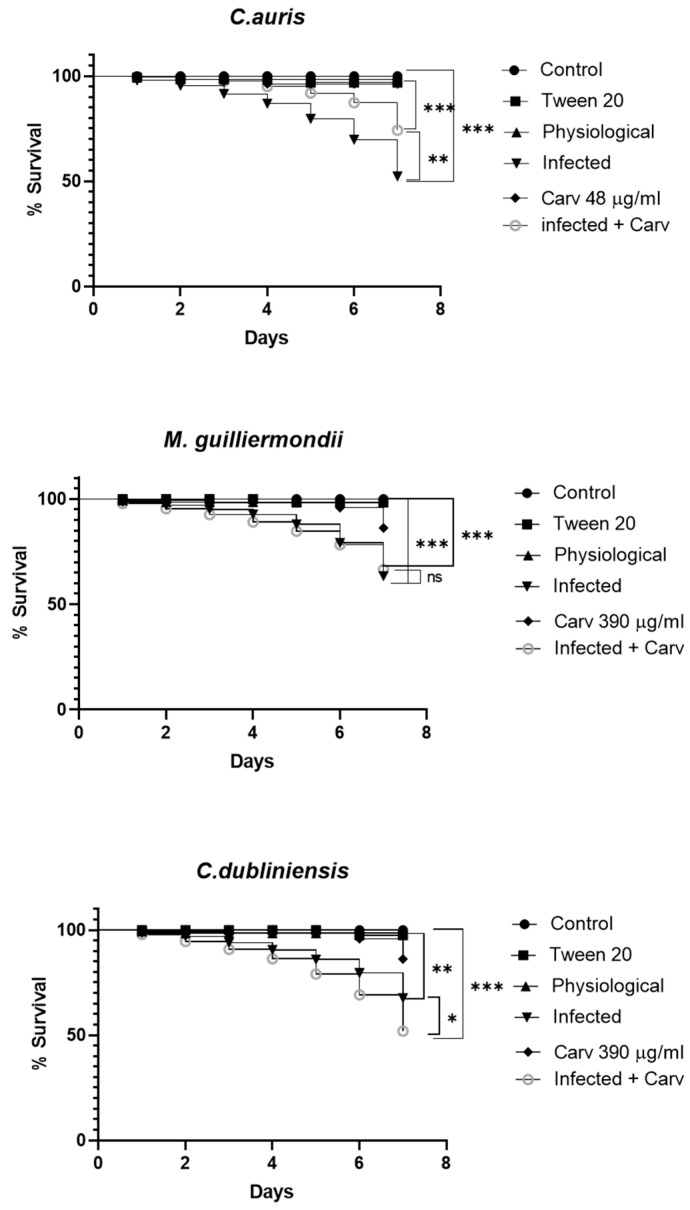
A survival test was conducted on *G. mellonella*, both with and without treatment with carvacrol at the respective MIC concentrations for *Candida auris*, *Meyerozyma guilliermondii* and *C. dubliniensis.* Ten larvae were used per group, each injected with 10 µL in the right proleg. Survival was monitored every 24 h for 7 days. Control: larvae untreated; Physiological: larvae treated with Physiological solution; Infected: larvae infected with 10^7^ cell/mL; Carv: larvae treated only with MIC concentration carvacrol; *C. auris* 48 μg/mL, *M. guilliermondii* 390 μg/mL and *C. dubliniensis* 390 μg/mL Infected + Carv, larvae infected with 10^7^ cell/mL and 1 h post infection treated with carvacrol. Results are expressed as % survival in comparison to uninfected (PBS-treated) larvae. Median values obtained per group (10 larvae) are presented. For survival rates, the log-rank (Mantel-Cox) test method was used. (* *p* < 0.05. ** *p* < 0.01, *** *p*< 0.001, ns = not significant *p* > 0.05).

The viability of *G. mellonella* larvae infected with *C. auris*, *M. guilliermondii* and *C. dubliniensis* with and without carvacrol treatment is shown in Figure 3. As reported in a previous paper, carvacrol was not toxic at the MIC concentration, and the survival rate was similar to that of the controls [21]. After seven days, the mortality rate of the larvae infected with about 5 × 10^5^ cells of all three clinical isolates was approximately 50%. The presence of carvacrol at the MIC concentration increased survival by approximately 20% (** *p* < 0.01), but only in larvae infected with *C. auris*. No increase in survival was observed in larvae infected with *M. guilliermondii* while increased mortality was observed in larvae infected with *C. dubliniensis* (* *p* < 0.05).

### 3.5. Osmotic Stress in Presence of Carvacrol

**Figure 4:**
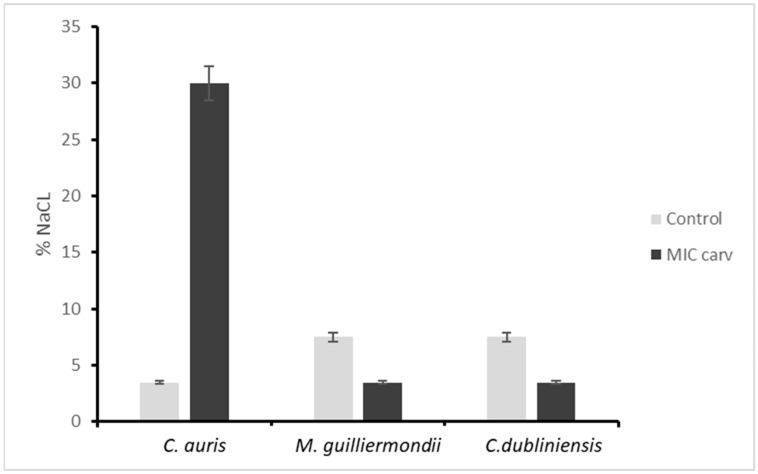
Histograms showing the growth of *C. auris*, *M. guilliermondii* and *C. dubliniensis* in the presence of different concentrations of NaCl, with or without the MIC concentration of carvacrol. Control: cell growth in SD medium with % NaCl added from 0.03 to 30%. MIC Carv: cells grown in SD medium with % NaCl from 0.03 to 30%, plus the MIC concentration of carvacrol. *C. auris* 48 μu/mL, *M. guilliermondii* 390 μg/mL and *C. dubliniensis* 390 μg/mL.

Yeast cells were inoculated in SD broth with a gradual increase in NaCl concentration to assess resistance to osmotic stress, both with and without MIC concentrations of carvacrol, as shown in Figure 4. *C. auris* isolate were able to grow at a concentration of 3.75% NaCl, while *M. guilliermondii* and *C. dubliniensis* were able to grow at 7.5% NaCl in the control group without carvacrol, demonstrating partial resistance to osmotic stress. In the presence of the MIC concentration of carvacrol, only *C. auris* was able to grow at a concentration of 30% NaCl, demonstrating increased resistance to osmotic stress; the other two genera were unable to grow in the presence of a high concentration of NaCl.

### 3.6. Cytotoxicity Assays on Human Cell Lines

**Figure 5:**
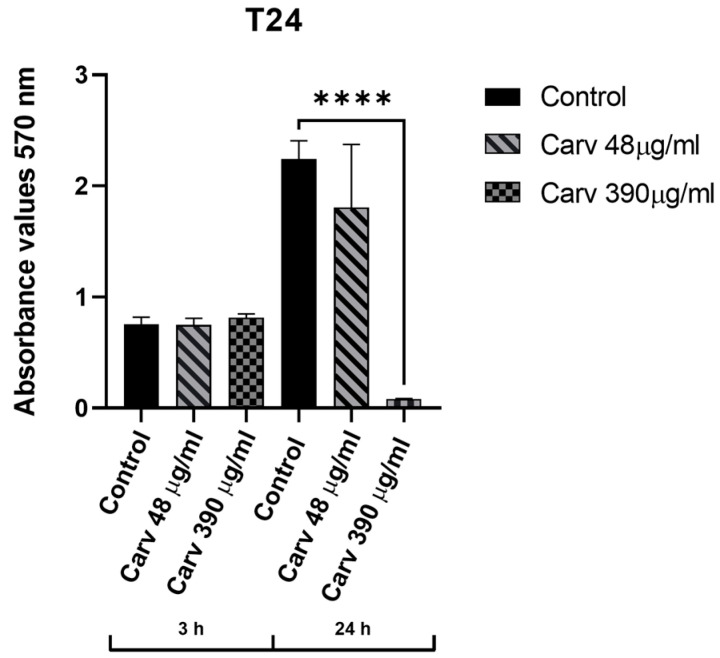
MTT assay on T24 cell lines treated with carvacrol at 48 µg/mL and 390 µg/mL, after 3 h and 24 h of treatment. Control: cell growth in RPMI added with 10% of FBS, evaluated after 3 h and 24 h of treatment. Carv 48 µg/mL: cell growth in RPMI added with 10% of FBS and carvacrol concentration of 48 µg/mL, evaluated after 3 h and 24 h of treatment. Carv 390 µg/mL: cell growth in RPMI added with 10% of FBS and carvacrol concentration of 48 µg/mL, evaluated after 3 h and 24 h of treatment (**** *p* < 0.0001).

The cytotoxic activity of carvacrol was tested on T24 cell lines using the MTT assay. After 3 h of contact the concentrations of carvacrol of 48 µg/mL and 390 µg/mL did not have a significant impact on cell viability in the T24 cell line used (Figure 5). Following a 24-h exposure period at a concentration of 48 µg/mL, no statistically significant reduction in cell viability was observed, whereas a reduction was observed at a concentration of 390 µg/mL.

## 4. Discussion

The growing incidence of infections caused by NAC species and the increasing antifungal resistance among clinical isolates was recognized as one of the most important public health threats of the 21st century and emphasizes the need for alternative therapeutic strategies [15]. In this context, natural compounds such as the natural monoterpene phenol, carvacrol, emerge as promising alternatives, because of their antifungal properties and their ability to modulate key virulence factors. Our study evaluated the antifungal activity and antivirulence effects of the carvacrol, against *C. auris*, *M. guilliermondii* and *C. dubliniensis* since its antimicrobial activity against bacteria and fungi has been proved [43]. Our results demonstrate a significant modulatory effect of carvacrol on several virulence traits, confirming its potential role as a promising antifungal and antivirulence agent.

The MIC value of carvacrol against *C. auris* obtained in our study was lower and it differs from the higher MIC values reported by other authors [44]. Reginato et al. [45] reported similar antifungal activity for carvacrol against *C. dubliniensis*, with an MIC range of 78–625 μg/mL. Similar results were reported for *M. guilliermondii*, with a MIC value between of 256 and 512 μg/mL [46].

Adherence is the first step in host colonization and depends on hydrophobic interactions on the microorganism surface. These two factors could be key targets for reducing pathogenicity. An important feature of *C. auris* that is involved in adhesion processes is its distinctive cell wall mannosylation, which differs from that of other genera [47]. This could explain the substantial difference observed compared to the treatment of other species, when carvacrol is present. Carvacrol can disrupt the cell wall mannosylation process of *C. auris*. By impacting mannosylation, carvacrol could indirectly affect fungal cell wall integrity, adhesion, and host immune response, which are all functions linked to mannose residues in the cell wall. 

Biofilm development is a critical virulence trait that enables fungal persistence and resistance to antifungal agents [48]. Carvacrol has been shown to significantly reduce biofilm formation in *M. guilliermondii* and *C. dubliniensis*, while its effects on *C. auris* were more limited. Notably, the marked (>80%) reduction of preformed biofilms in *M. guilliermondii* and *C. dubliniensis* underscores carvacrol’s potential to prevent and disrupt biofilm development. This finding is consistent with the results reported by Miranda-Cadena et al. [23] and Shaban et al. [49].

Although *C. auris* biofilms exhibited greater resilience to carvacrol treatment, a statistically significant reduction in preformed biofilms was observed after 48 h. This delayed effect is consistent with previous reports that *C. auris* biofilms possess a dense extracellular matrix and altered metabolic profiles, contributing to high antifungal tolerance [50]. The modest yet significant reduction in biofilm metabolism, coupled with the increased survival of *G. mellonella* larvae infected with *C. auris* and treated with carvacrol, supports the hypothesis that carvacrol has the potential to reduce pathogenicity, even in multidrug-resistant strains [27].

Several studies have examined the use of EO, either alone or in combination with synthetic drugs, to inhibit *C. auris* biofilm formation [50]. However, little research has been conducted on the inhibition of *C. auris*, *M. guilliermondii* and *C. dubliniensis* biofilm formation in the presence of substances such as carvacrol. Nevertheless, some authors have reported reduced adhesion to human cells in the presence of carvacrol in *C. auris* [48]. The present study demonstrates that the cellular viability of human cells treated with carvacrol for a period of three hours is not compromised. As demonstrated in preceding studies, treatment of human cell lines with carvacrol over a duration of 24 h has been shown to result in toxicity, particularly in instances where elevated concentrations are employed [41]. As demonstrated in previous studies, the synergistic effect of this substance with antifungals enables lower concentrations of both substances to be used, achieving non-toxic concentrations on cell lines [41]. This finding indicates a necessity for new formulations for administration to be sought.

The differential impact of carvacrol on CSH and adherence suggests potential species-specific interactions. Carvacrol significantly inhibited adherence in *M. guilliermondii* and *C. dubliniensis*, likely contributing to their reduced biofilm formation. Interestingly, an increase in CSH was observed in *M. guilliermondii* following treatment, which could suggest a compensatory adaptation or membrane remodeling effect, as proposed by De Groot et al. [34]. In contrast, *C. auris*, which already exhibits higher baseline hydrophobicity, showed no significant modulation, possibly due to its inherently robust surface architecture and ability to persistently colonize [5].

The in vivo *G. mellonella* model provided further insight into the therapeutic potential of carvacrol. The increased survival of the larvae infected with *C. auris* and treated with carvacrol is encouraging, suggesting that modulation of virulence may provide therapeutic benefits, even in the absence of strong fungicidal activity, given the clinical challenges posed by this pathogen [9]. However, the increased mortality of larvae infected with *C. dubliniensis* in the presence of carvacrol highlights the need to investigate possible species-specific interactions, host immune modulation, or altered fungal metabolism.

Another notable finding was the enhanced osmotic stress tolerance of *C. auris* when exposed to carvacrol, suggesting a potential adaptive response of this species. The activation of stress-response pathways is a well-recognized feature of *C. auris* and may contribute to its survival in adverse environments as well as its persistence on hospital surfaces, including those involving osmotic and oxidative stress [17].

Taken together, our results suggest that carvacrol acts not only as a direct antifungal compound but also as a modulator of critical virulence traits.

Although its fungicidal activity was weaker than that of fluconazole, its impact on adherence, biofilm formation and survival in vivo supports its potential as an adjuvant therapy to conventional antifungals, particularly against these species. By targeting virulence rather than viability, carvacrol could reduce selective pressure and help limit antifungal resistance.

Future research should explore the synergistic effects of combinations of carvacrol with clinically used antifungals and investigate the molecular mechanisms behind the observed phenotypic modulatory effects and modulatory effects.

## 5. Conclusions

Carvacrol shows moderate fungicidal activity and demonstrates a strong modulatory effect on virulence factors in NAC species. It significantly reduces adherence and biofilm formation in *M. guilliermondii* and *C. dubliniensis*, while enhancing survival in a *C. auris* infection model. These findings support the potential role of carvacrol as an antifungal adjuvant, which is particularly relevant in the current context of rising antifungal resistance.

Focusing on virulence traits rather than fungal viability represents an innovative strategy to minimize selective pressure and the emergence of resistance.

Further research is required to elucidate the molecular mechanisms involved and to confirm the clinical relevance of these effects in advanced preclinical models.

## Figures and Tables

**Table 1:** Antifungal activity of fluconazole and carvacrol against *Candidozyma auris*, *Meyerozyma guilliermondii* and *Candida dubliniensis*.

	Fluconazole (μg/mL)	Carvacrol (μg/mL)
	MIC	MIC
IMR-M-L 1304 *C. auris*	4	48
IMR-M-L 1511 *M. guilliermondii*	32	390
IMR-M-L 1510 *C. dubliniensis*	0.5	390

MIC = Minimum Inhibitory Concentration.

## Data Availability

The original contributions presented in this study are included in the article/Supplementary Materials. Further inquiries can be directed to the corresponding author.

## Supplementary Materials

The following supporting information can be downloaded at https://www.mdpi.com/article/10.3390/microorganisms14010188/s1, Figure S1. Histograms showing the average weight of the larvae in each single group. Control: cells not treated; Tween 20: cells treated with 0.01% of Tween 20 in physiological saline; Infected + Carv, larvae infected with 107 cell/mL and 1 h post infection treated with carvacrol. Carv: larvae treated only with MIC concentration carvacrol; Infected: larvae infected with 107 cell/mL. The results represent the means ± standard deviation from three independent experiments. *p*-values were obtained using a Student’s *t*-test (* *p* < 0.05); ns = no significate *p* > 0.05.
