# Supplementary figures and images for: Carvacrol Reduces Virulence Traits in Meyerozyma guilliermondii and Candida dubliniensis and Enhances Galleria mellonella Survival During Candidozyma auris Infection
Source: Microorganisms. 2026 Jan 14;14(1):188. doi: 10.3390/microorganisms14010188 (PMC12843738; doi:10.3390/microorganisms14010188)

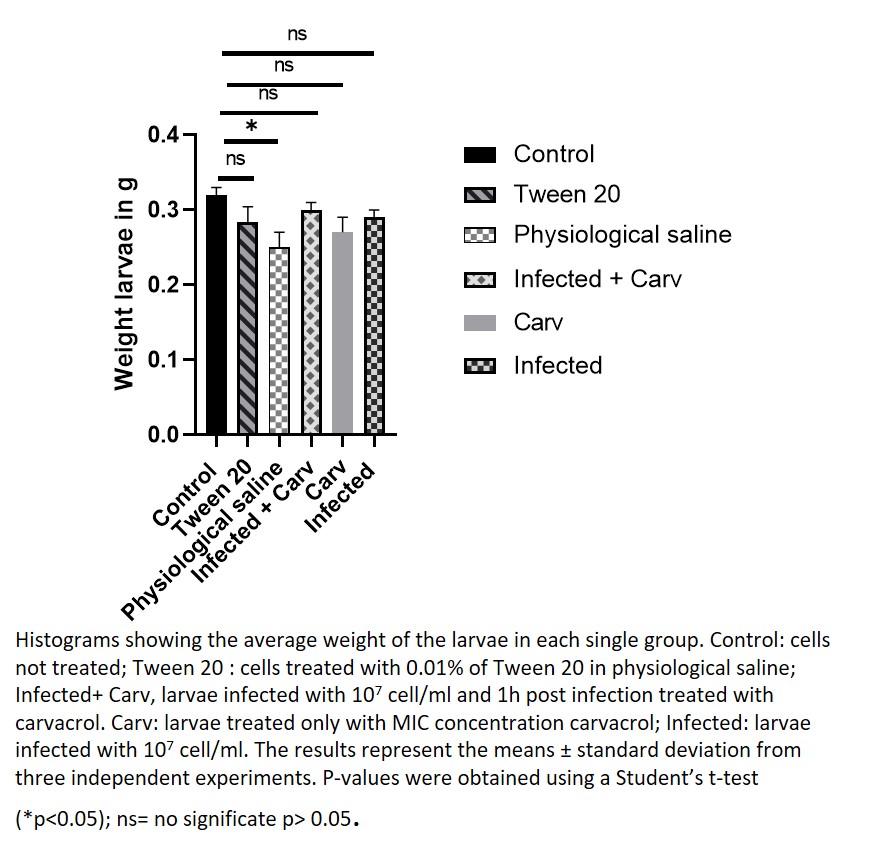

Supplement: Supplementary file 1 [file microorganisms-14-00188-s001.zip › microorganisms-3908961-supplementary.jpg]
